# Epidemiological and genomic characteristics of global *mcr*-positive *Escherichia coli* isolates

**DOI:** 10.3389/fmicb.2022.1105401

**Published:** 2023-01-18

**Authors:** Jiping Shi, Hong Zhu, Chang Liu, Hui Xie, Chuchu Li, Xiaoli Cao, Han Shen

**Affiliations:** ^1^Department of Laboratory Medicine, Nanjing Drum Tower Hospital, The Affiliated Hospital of Nanjing University Medical School, Nanjing, Jiangsu, China; ^2^Department of Acute Infectious Disease Control and Prevention, Jiangsu Provincial Center for Disease Control and Prevention, Nanjing, China

**Keywords:** *Escherichia coli*, *mcr*, whole genome sequencing, antimicrobial resistance genes, sequence types

## Abstract

**Objective:**

The worldwide dissemination of colistin-resistant *Escherichia coli* (*E. coli*) endangers public health. This study aimed to better understand the global genomic epidemiology of *E. coli* isolates carrying mobilized colistin resistance (*mcr*) genes, providing information to assist in infection and prevention.

**Methods:**

*Escherichia coli* genomes were downloaded from NCBI, and *mcr* was detected using BLASTP. Per software was used to extract information on hosts, resources, collection data, and countries of origin from GenBank. Sequence types (STs), prevalence of plasmids, antimicrobial resistance genes (ARG), and virulence factors (VF) in these genomes were analyzed. Statistical analyses were performed to assess the relationships between *mcr*, ARGs, plasmids, and STs.

**Results:**

In total, 778 *mcr*-positive isolates were identified. Four *mcr* variants were detected, with *mcr*-1 (86.1%) being the most widespread, followed by *mcr*-9 (5.7%), *mcr*-5 (4.4%), and *mcr*-3 (3.0%). Multiple ARGs were identified, with *bla*_CTX–M_ (53.3%), *fosA* (28.8%), *qnr* (26.1%), *bla*_*NDM*_ (19.8%), and *aac (6’)-Ib-cr* (14.5%) being the most common. Overall, 239 distinct STs were identified, of which ST10 (13.8%) was the most prevalent. A total of 113 different VFs were found, *terC* (99.9%) and *gad* (83.0%) were most frequently detected. Twenty types of plasmids were identified; IncFIB (64.1%), IncX (42.3%), and IncX (42.3%) were the most common replicons. IncI2 and IncX4 were frequently detected in *mcr*-1-positive isolates, whereas IncFII, IncI1-I, and IncHI2 were dominant plasmids in *mcr*-3, *mcr*-5, and *mcr*-9-positive isolates, respectively. A higher frequency of ARGs and VFs was observed among ST156 and ST131 isolates.

**Conclusion:**

Our data indicated that more than half of the *mcr*-positive *E. coli* strains carried endemic ARGs and VFs. ST10 and ST156 isolates deserved further attention, given the rapid transmission of ST10 and the convergence of ARGs and VFs in ST156.

## Introduction

*Escherichia coli* is a common pathogen responsible for multiple infections, including those in the urinary tract, bloodstream, and wounds, in humans and animals worldwide (Nang et al., 2019). It can easily acquire multi-drug resistance, owing to its ability to accumulate multiple resistance genes, primarily through horizontal gene transfer of mobile elements. Currently, its acquisition of genes encoding extended-spectrum β-lactamases (ESBLs), carbapenem-hydrolyzing ß-lactamase (CHßls), 16S rRNA methylases, plasmid-mediated quinolone resistance (PMQR), and plasmid-mediated glutathione S-transferase (PMGST) is of great clinical concern (Simoni et al., 2021; Yin et al., 2021; Guo et al., 2022), accelerating the evolution of drug resistance, making colistin the last-resort antibiotic for treating infections caused by multi-drug resistant bacteria. The use of colistin in livestock as a growth promoter has been banned in China (Wang et al., 2020) due to increasing polymyxin resistance. However, the use of colistin in clinical settings is increasing because of the emergence of increased multi-drug resistance (MDR), particularly carbapenem-resistant *E. coli* (*CRE*) (Liu et al., 2020); therefore, the emergence and spread of colistin-resistant *E. coli* are inevitable.

Colistins primarily bind to the outer membrane lipopolysaccharide through the interaction of their cationic residues with the phosphate groups of lipid A, changing the permeability of the cell envelope, leading to leakage of cell contents and bacterial death (Rodríguez-Santiago et al., 2021). However, mobile colistin resistance (*mcr*) genes have been reported to be the primary mechanism conferring resistance to colistin, although two-component systems (TCSs), such as PmrAB and PhoPQ; mutation(s) in the MgrB regulator; LPS modification (Bialvaei and Samadi Kafil, 2015); LPS overall charge changes, and the reduced affinity of colistin for the outer membrane, also contribute to colistin resistance based on chromosomal-encoded mechanisms (Zhang et al., 2021; Phuadraksa et al., 2022). The *mcr* genes encode transferable phosphoethanolamine transferases that modify the lipopolysaccharide of the bacterial outer membrane to weaken its binding to colistin (Yin et al., 2021). To date, more than 10 *mcr* variants (*mcr*-1 to *mcr*-10) have been identified, carried on different conjugative and non-conjugative plasmid backbones (Mmatli et al., 2022). These variants have been widely identified in many bacteria, including *E. coli*, *Klebsiella*, *Enterobacter*, *Citrobacter*, *Proteus*, *Providencia*, *Salmonella*, *Pseudomonas*, *Acinetobacter*, *Aeromonas*, *Kluyvera*, and *Raoultella*, as well as in diverse ecosystems, including soil, botanicals, wildlife, animal environments, and public places (Nang et al., 2019; Anyanwu et al., 2020). However, despite its status as the most common host for *mcr* genes, data on *mcr* distribution in *E. coli* are quite limited, although global surveillance showed an approximately 1.26% prevalence of *mcr*-positive *E. coli* among humans, animals, and environments (Dadashi et al., 2022; Mmatli et al., 2022), with the *mcr*-1, *mcr*-3, *mcr*-5, *mcr*-9, and *mcr*-10 variants most prevalent (Dadashi et al., 2022). These investigations all relied on PCR detection methods, with few data on the prevalence of *mcr* from global genomic databases.

Most importantly, *mcr* genes are carried by mobile genetic elements, especially conjugative plasmids, leading to their broad dissemination and establishment worldwide under the selective pressure of antimicrobial agents. Although isolates with *mcr* as well as other antimicrobial resistance genes (ARG), such as *bla*_CHß*Ls*_, *bla*_ESBLs_, PMQR, and *fosA*, have been frequently reported, posing clinical challenges (Haenni et al., 2016; Zhang W. et al., 2022), the prevalence of clinically endemic ARGs among *mcr*-positive strains remains unclear. Furthermore, a high diversity of plasmid reservoirs has been shown to be associated with *mcr* genes (Nang et al., 2019); however, the distribution of plasmid replicons among global *mcr*-positive *E. coli* and the associations between *mcr* and plasmid replicons remain unknown. Furthermore, multiple virulence factors (VFs) involved in bacterial adherence, invasion, immune modulation, effector delivery systems, and nutritional/metabolic factors have been identified in *E. coli* (Zhao et al., 2020; Kubelová et al., 2021). However, their distribution among *mcr*-positive *E. coli* and the association between VFs and ARGs require further investigation. Finally, the prevalence of VFs among internationally popular sequence types (STs) of *mcr*-positive *E. coli* is of vital importance. All of these will contribute to optimizing prevention and control measures to prevent further spread and outbreaks of *mcr*-positive infections.

In this study, we first investigated the distribution of *mcr* among global *E. coli* based on the whole genome sequencing (WGS) data from GenBank. We analyzed STs and the prevalence of ARGs, VFs, and plasmid replicons. Additionally, the distribution consistency of VFs and ARGs was tested, providing epidemiological data for the implementation of infection and prevention.

## Materials and methods

### Genomes

All *E. coli* genomes updated to 2021 were downloaded from NCBI using Aspera software in batches (Kim et al., 2018). As some of the genome-wide sequencing data from 2019 to 2021 have not yet been released when the study started, only strains identified from 1905 to 2019 were included in this study. Totally, 22,884 genomes were downloaded and tested for quality. The qualifying parameters were completeness >90% and contamination <5, with contig quantity ≤500 and N50 ≥40,000.

### *mcr* identification

All *mcr* sequences were obtained from the NCBI Biological Resistance Reference Gene Database [(*mcr*)].^1^ BLASTP was performed, with thresholds set as expected value = 1e-5, coverage ≥50%, identity = 100%, and matching length = subject gene length.

### Phylogenetic tree construction

Prokka was used to annotate the 778 *E. coli* isolates, and Roary software was adopted to obtain multiple sequence alignment files of 1,222 core genes (Zhou et al., 2021), which were further used to acquire the single nucleotide polymorphism information through SNP sites. Finally, RaxML was used to construct the maximum likelihood tree, and the evolution tree results were visualized by itol (Liu et al., 2022).

### Sequence types

Sequence types of *mcr*-positive *E. coli* were identified using CLC workbench version 21.0.1. After consensus sequences were extracted, STs were analyzed using multilocus sequence typing (MLST) with *E. coli* (Oxford) as the reference database.

### Antimicrobial resistance prevalence

The distribution of other ARGs was investigated using CLC workbench version 21.1. The fasta file was input into the files in CLC data using standard import, and after the consensus sequence was extracted, the prevalence of ARGs was analyzed using the ResFinder database for comparison. The results were exported as scv files, and further sorted for analysis.

### Distributions of plasmid replicons and VFs

Genomes were submitted to the Center for Genomic Epidemiology. PlasmidFinder software^2^ was used to identify plasmid replicons, and Virulence Finder 2.0^3^ was used to identify VFs.

### Statistical analyses

Correlation analyses were performed using IBM SPSS 22.0. The distribution consistency of VFs and ARGs was tested by McNimar analysis, and a *p*-value of > 0.05 was taken as the consistency between them.

## Results

### General characteristics

**FIGURE 1:**
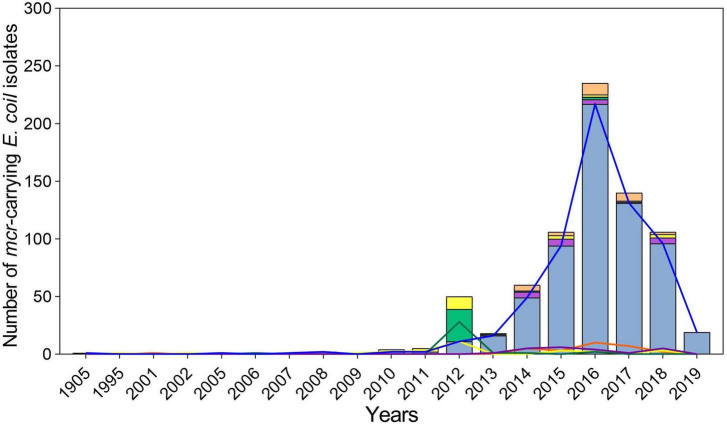
Characteristics of global spread of mcr-positive *Escherichia coli* per year. The light blue columns and blue lines represent mcr-1, the mauve columns and purple lines represent mcr-3, the light green columns and green lines represent mcr-5, the yellow columns and lines represent mcr-9, and the orange columns and lines represent the combination of varied mcr genes.

Overall, 778 *mcr*-positive *E. coli* isolates were identified from 22,884 *E. coli* genomes (Supplementary Table 1). They were isolated in 25 countries covering 6 continents: including Asia (*n* = 555), South America (*n* = 119), Europe (*n* = 40), North America (*n* = 27), Africa (*n* = 17), and Oceania (*n* = 13). China (*n* = 401), Japan (*n* = 26), Thailand (*n* = 41), Laos (*n* = 22), Brazil (*n* = 37), Paraguay (*n* = 28), and the USA (*n* = 25) were the most common sources. From 1905 to 2011, only 13 *mcr*-positive isolates were submitted (Figure 1). The number of *mcr*-positive isolates gradually increased from 2012; and hundreds of genomes were submitted every year peaking in 2016 (*n* = 235).

Regarding sources, we found that animals (*n* = 347, 44.6%) were the most common, with chickens (*n* = 202), pigs (*n* = 44), and cattle (*n* = 32) being the most common species. Feces (*n* = 72), cecum/rectal/cloaca swabs (*n* = 37), and meat (*n* = 33) were the dominant sources (Table 1). Humans accounted for 38.8% (*n* = 302) of all isolates taken from feces (*n* = 71), urine (*n* = 68), rectal swabs (*n* = 39), gut (*n* = 34), and blood (*n* = 20) (Table 1). Notably, *mcr*-positive strains were also found in the environment (*n* = 56, 7.2%), such as farms, hospitals, foods, and water.

**TABLE 1:** The hosts and sample types of mcr-positive *Escherichia coli* isolates worldwide.

Hosts (*n*)^a^		Sample types (*n*)
Animals (347)	Chicken (195)	Cecum/rectal/cloaca swabs (37), chicken meat (33), feces (31), lung (1), carcass (1), NA (92)
	Pig (44)	Feces (16), rectal swab (9), nares swab (3), cecum (3), meat (3), liver (1), NA (9)
	Cattle (26)	Feces (19), NA (7)
	Fly and gull (30)	Cloacal swabs (11), feces (6), NA (13)
	Waterfowl (22)	NA (22)
	Canine (11)	Blood (3), anal swab (1), prostatic wash (1), NA (6)
	Others (12)	Gallopavo sample (4), Fish sample (4), Giant Panda feces (1), horse sample (1), spheniscus foot lesion (1), vulpes zerda feces (1)
Humans (302)		Feces (71), urine (68), rectal swab (39), gut (34), blood (20), respiratory secretions (12), ascites (4), abdomen incision swab (3), foot/leg secretion (3), bile (2), bone (1), soft t*iss*ue (1), vaginal secretion (1), other body fluids/secretion/pus (6), NA (37)
Environments (56)		Chicken/pig/dairy cattle farms (12), animal food (10), hospital (6), vegetable (5), laboratory (4), seawater (5), wastewater (3), coastal water/estuarine (2), drinking water (2), raw milk cheese (4), NA (3)

^a^Among the 778 strains, only 705 strains indicate host and sample type. NA, not applicable.

### *mcr* variant distribution

**FIGURE 2:**
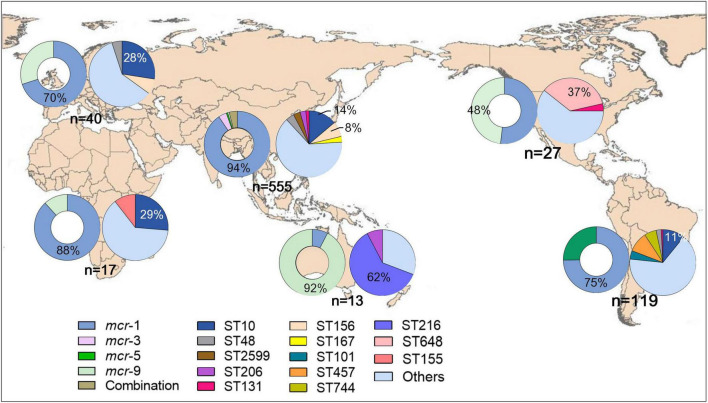
Geographical distribution of mcr and STs of mcr-positive *Escherichia coli* worldwide. The hollow and solid pie charts of each continent represent the distribution of mcr and STs, respectively.

Four *mcr* variants were identified among the 778 isolates, with *mcr*-1 (*n* = 654, 86.1%) being the most prevalent one, followed by *mcr*-9 (*n* = 44, 5.7%), *mcr*-5 (*n* = 34, 4.4%), and *mcr*-3 (*n* = 23, 3.0%), besides, two or more *mcr* variants were found in 23 isolates. For subtypes, 1.1 (*n* = 633, 96.8%) was the most prevalent for *mcr*-1, with subtypes 1.5 and 1.7 also observed, accounting for 0.9% (*n* = 6) and 1.5% (*n* = 10) of *mcr*-1, respectively. Subtypes 3.1, 3.4, and 3.5 were common variants of *mcr*-3, accounting for 56.5% (*n* = 13), 13.0% (*n* = 3), and 21.7% (*n* = 5), respectively. No subtypes of *mcr*-5 and *mcr-*9 were detected. Notably, *mcr*-1, *mcr*-5, and *mcr*-9 were first identified in isolates collected in 1905, 1995, and 2001, respectively, before colistin was produced and used in veterinary and clinical settings. The *mcr*-3 variant emerged in 2013 and has begun to spread more recently (Figure 1). Geographically, Oceania variants were mostly *mcr*-9 (*n* = 12, 92.3%), whereas the most prevalent variants in South America were *mcr*-1 (*n* = 89, 74.8%) and *mcr*-5 (*n* = 30, 25.2%) (Figure 2). Both *mcr*-1 and *mcr*-9 were endemic in Europe, North America, and Africa, with *mcr*-1 accounting for 51.9%–88.2%. The *mcr*-1 (*n* = 523, 94.2%) and *mcr*-3 (*n* = 23, 4.1%) variants were most common in Asia (Figure 2).

### Genetic relationship

**FIGURE 3:**
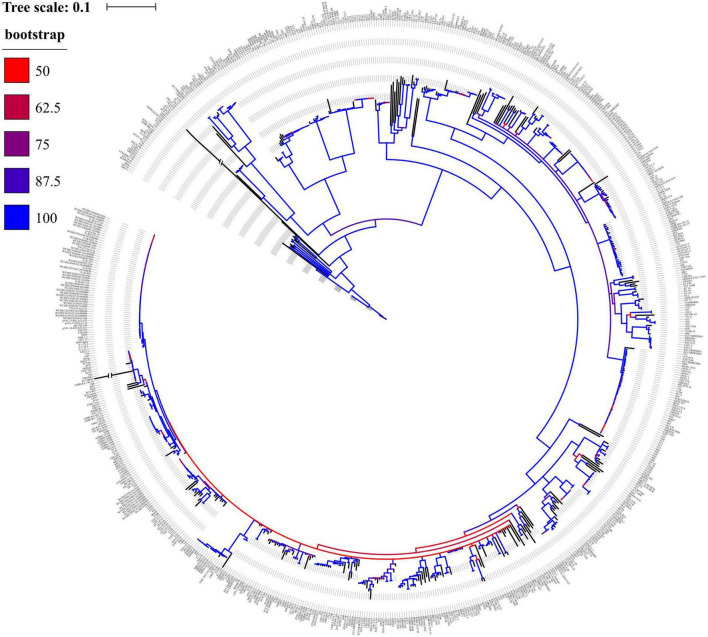
Phylogenetic tree of 778 mcr-positive *Escherichia coli* isolates.

Overall, the phylogenetic tree displayed that 778 *mcr*-positive isolates were divided into 292 clades (Figure 3), indicating a genetic diversity of such strains. Notably, the biggest clade was composed of 55 isolates, of which 76.4% (*n* = 42) were ST10 from China and Colombia. Another large clade was composed of 48 isolates and 60.4% (*n* = 29) of them were ST156 from China, suggesting clonal dissemination of ST10 and ST156 mainly in China.

### Antimicrobial resistance determinants

**FIGURE 4:**
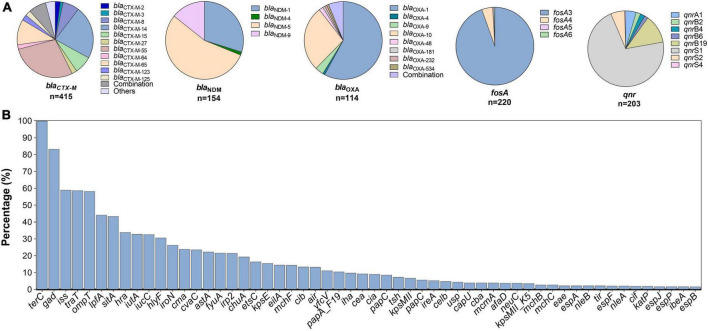
Subtypes of antimicrobial resistance genes **(A)** and prevalence of common virulent factors **(B)** among mcr-positive *Escherichia coli*.

Multiple ARGs were identified in *mcr*-positive strains. The most common were ESBLs *bla*_CTX–M_ (*n* = 415, 53.3%) and *bla*_OXA_ (*n* = 114, 14.7%); CHßLs *bla*_NDM_ (*n* = 154, 19.8%) and *bla*_KPC_ (*n* = 15, 1.9%); pAmpCs *bla*_CMY_ (46, 5.9%) and *bla*_DHA_ (*n* = 7, 0.9%); 16s rRNA methylases *rmtB* (*n* = 88, 11.3%) and *armA* (*n* = 6, 0.8%); PMQRs *aac (6’)-Ib-cr* (*n* = 113, 14.5%), *oqxAB* (*n* = 56, 7.2%), and *qnr* (203, 26.1%); and the fosfomycin resistance gene *fosA* (*n* = 220, 28.3%) were detected. The common variant subtypes are shown in Figure 4A; *bla*_CTX–M–55_ (*n* = 117, 28.2%), *bla*_CTX–M–14_ (*n* = 94, 22.7%), and *bla*_CTX–M–65_ (*n* = 45, 10.8%) were dominant in *bla*_*CTX–M*_, whereas *bla*_NDM–5_ (*n* = 84, 54.6%), *bla*_*NDM–*1_ (*n* = 47, 30.5%), and *bla*_NDM–9_ (*n* = 19, 22.6%) were the most prevalent in *bla*_NDM_.

### Virulence factors

In total, 112 different VFs were identified, *terC* (*n* = 777, 99.9%) and *gad* (*n* = 646, 83.0%) being the most prevalent (Figure 4B). VFs involved in intestinal invasiveness and anti-phagocytosis, including *sitA*, *iss*, and *iroN*, were carried by 43.7% (340/778), 59.0% (459/778), and 26.4% of all isolates, respectively. Approximately 50% of the isolates contained *traT*, *ompT*, and *lpfA* with specific carrying rates of 58.61% (456/778), 57.84% (450/778), and 43.96% (342/778), respectively. Other VFs, such as *hra* (*n* = 264, 33.9%), *iutA* (*n* = 256, 32.9%), *iucC* (*n* = 256, 32.9%), and *hlyF* (*n* = 239, 30.7%), were also frequently detected. Other relatively less common VFs are shown in Figure 4B.

### Sequence types

Overall, 239 distinct STs were identified, with ST10 (*n* = 107) being the most frequent. ST156 (*n* = 46), ST48 (*n* = 29), ST457 (*n* = 26), ST648 (*n* = 22), ST167 (*n* = 19), ST2599 (*n* = 19), ST101 (*n* = 18), ST206 (*n* = 16), ST617 (*n* = 15), ST744 (*n* = 13), ST155 (*n* = 12), ST2705 (*n* = 12), ST354 (*n* = 12), ST216 (*n* = 11), ST131 (*n* = 11), and ST69 (*n* = 10) were also detected. Other STs were detected (*n* < 10). Geographically, ST10 was the dominant type in Asia, Africa, Europe, and South America; ST156 was endemic in both Asia and Europe; and ST457 was the most common type in South America (Figure 2). The most common STs in Oceania and North America were ST216 and ST648, respectively (Figure 2).

### Plasmid replicons

Of the 778 isolates, 20 types of plasmid replicons were identified with IncFIB (*n* = 499, 64.1%) being the most common replicon, followed by IncX (*n* = 329, 42.3%), IncFII (*n* = 316, 40.6%), IncI2 (*n* = 300, 38.6%), IncHI2 (*n* = 257, 33.0%), Col (*n* = 177, 22.8%), IncP (*n* = 210, 27.0%), IncFIA (*n* = 186, 23.9%), IncFIC (*n* = 150, 19.3%), IncI1-I (*n* = 179, 23.0%), IncY (*n* = 105, 13.5%), IncN (*n* = 170, 21.9%), and other more rare plasmid replicons (Table 2). Despite 50–70% of strains carrying IncFIB plasmids, several plasmids, such as IncI2, IncX, IncHI2, and IncFII, were relatively highly carried in *mcr-*1 strains, whereas plasmid IncFII was common in *mcr*-3 strains; 90.9% of *mcr*-9 strains carried IncHI2 and more than 70% of *mcr*-5 positive strains carried IncFII and IncI1-I plasmids (Table 2). The distribution of *bla*_NDM_ was correlated with those of the plasmid replicons IncFIA, Col, IncI1-I, and IncN (*p* > 0.05). The prevalence of *qnr* was correlated with those of IncFIA, Col, IncI1-I, and IncP (*p* > 0.05). Additionally, the incidences of *fosA*, IncFIA, and IncP were correlated (*p* > 0.05) (Table 3). No significant differences were found in the distribution of some VFs and plasmid replicons, including the combination of *hra*, *iutA*, *iucC*, *hlyF* with IncHI2; *sitA* with IncX; *lpfA* with IncFII; and *iroN* with IncFIA, Col, IncI1-I, IncN, and IncP (Table 3).

**TABLE 2:** Plasmids and their distributions among different mcr genes.

	IncFIA	Col	IncFIB	IncFII	IncHI2	IncI1-I	IncI2	IncN	IncP	IncX
*mcr*-1 (*n* = 654)	144 (22.0%)	153 (23.4%)	416 (63.6%)	254 (38.8%)	204 (31.2%)	144 (22.0%)	283 (43.3%)	141 (21.6%)	194 (29.7%)	302 (46.2%)
*mcr*-3 (*n* = 23)	8 (34.8%)	1 (4.4%)	16 (69.6%)	11 (47.8%)	8 (34.8%)	1 (4.4%)	1 (4.3%)	2 (8.7%)	2 (8.7%)	6 (26.1%)
*mcr*-5 (*n* = 34)	4 (11.8%)	3 (8.8%)	24 (70.6%)	28 (82.4%)	1 (2.9%)	25 (73.5%)	2 (5.9%)	12 (35.3%)	9 (26.5%)	1 (2.9%)
*mcr*-9 (*n* = 44)	19 (43.2%)	3 (6.8%)	24 (54.6%)	9 (20.5%)	40 (90.9%)	4 (9.1%)	5 (11.4%)	9 (20.5%)	1 (2.3%)	6 (13.6%)

**TABLE 3:** *P*-values for differences in distribution of plasmid replicons and resistant/virulent genes.

	IncFIA (*n* = 186)	Col (*n* = 177)	IncFIB (*n* = 499)	IncFII (*n* = 316)	IncHI2 (*n* = 257)	IncI1-I (*n* = 179)	IncI2 (*n* = 300)	IncN (*n* = 170)	IncP (*n* = 210)	IncX (*n* = 329)
*bla*_CTX–M_ (*n* = 415)	0.000	0.000	0.000	0.000	0.000	0.000	0.000	0.000	0.000	0.000
*bla*_NDM_ (*n* = 154)	0.067	0.151	0.000	0.000	0.000	0.14	0.000	0.333	0.000	0.000
*bla*_OXA_ (*n* = 114)	0.000	0.000	0.000	0.000	0.000	0.000	0.000	0.000	0.000	0.000
*Aac (6’)-Ib-cr* (*n* = 113)	0.000	0.000	0.000	0.000	0.000	0.000	0.000	0.000	0.000	0.000
*oqxAB* (*n* = 56)	0.000	0.000	0.000	0.000	0.000	0.000	0.000	0.000	0.000	0.000
*floR* (*n* = 403)	0.000	0.000	0.000	0.000	0.000	0.000	0.000	0.000	0.000	0.000
*qnr* (*n* = 203)	0.301	0.169	0.000	0.000	0.002	0.177	0.000	0.041	0.727	0.000
*fosA* (*n* = 220)	0.066	0.008	0.000	0.000	0.025	0.02	0.000	0.002	0.602	0.000
*rmtB* (*n* = 88)	0.000	0.000	0.000	0.000	0.000	0.000	0.000	0.000	0.000	0.000
*terC* (*n* = 777)	0.000	0.000	0.000	0.000	0.000	0.000	0.000	0.000	0.000	0.000
*gad* (*n* = 646)	0.000	0.000	0.000	0.000	0.000	0.000	0.000	0.000	0.000	0.000
*iss* (*n* = 459)	0.000	0.000	0.036	0.000	0.000	0.000	0.000	0.000	0.000	0.000
*traT* (*n* = 456)	0.000	0.000	0.005	0.000	0.000	0.000	0.000	0.000	0.000	0.000
*ompT* (*n* = 450)	0.000	0.000	0.006	0.000	0.000	0.000	0.000	0.000	0.000	0.000
*lpfA* (*n* = 342)	0.000	0.000	0.000	0.194	0.000	0.000	0.021	0.000	0.000	0.556
*sitA* (*n* = 340)	0.000	0.000	0.000	0.000	0.000	0.000	0.028	0.000	0.000	0.61
*hra* (*n* = 264)	0.000	0.000	0.000	0.006	0.744	0.000	0.039	0.000	0.003	0.001
*iutA* (*n* = 256)	0.000	0.000	0.000	0.001	1.000	0.000	0.014	0.000	0.013	0.000
*iucC* (*n* = 256)	0.000	0.000	0.000	0.001	1.000	0.000	0.014	0.000	0.013	0.000
*hlyF* (*n* = 239)	0.005	0.001	0.000	0.000	0.391	0.000	0.000	0.000	0.112	0.000
*iroN* (*n* = 205)	0.301	0.12	0.000	0.000	0.008	0.118	0.000	0.051	0.814	0.000

*p*-value of > 0.05 was taken as the consistency between plasmid replicons and resistant/virulent genes.

**FIGURE 5:**
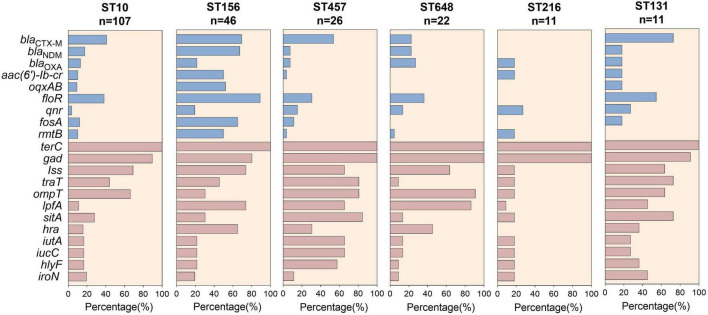
Carriage of antimicrobial resistance genes and virulent factors among globally prevalent ST strains.

The prevalence of ARGs and VFs among the ST10, ST156, ST457, ST648, ST216, and ST131 clones were compared. As the most prevalent ST worldwide, ST10 had a lower incidence of ARGs, whereas increased VFs and ARGs were observed among ST156 clones. For example, more than 50% of ST156 isolates carried *bla*_*CTX–M*_, *bla*_*NDM*_, *aac (6’)-Ib-cr*, *oqxAB*, *floR*, *fosA*, *rmtB*, *lpfA*, and *Hra* genes (Figure 4), indicating high virulence. Moreover, compared with ST648 and ST216, ST457 and ST131 were more commonly associated with VFs, including the *traT*, *ompT*, *lpfA*, *sitA*, *iutA*, *iucC*, *hlyF* genes, all of which have been implicated in bacterial survival, invasiveness, and adhesion (Figure 5).

## Discussion

As the last-line defense against MDR *E. coli* infections, colistin has been widely used to treat infections caused by extensive-drug resistant (XDR) bacteria, such as CRE, albeit it has been banned for use in livestock as a growth promoter. The clinical utility of colistin is currently threatened by the global dissemination of *mcr* and its co-occurrence with other endemic ARGs, such as ESBL and CHßl, through different plasmids. Thus, understanding the prevalent characteristics of global *mcr*-positive *E. coli* isolates, including the prevalence of STs, endemic ARGs, VFs, and plasmid replicons, will be useful to guide clinical treatment for serious infections, as well as the implementation of preventative measures.

In this study, we observed a rapid increase in the genomes of global *mcr*-positive *E. coli* from 2014 to 2016. Since 2016, many countries have prohibited colistin as a growth promoter for livestock, including Brazil (November 2016), Thailand (February 2017), China (April 2017), Japan (July 2018), Malaysia (January 2019), Argentina (February 2019), and India (July 2019) (Center for Veterinary Drug Development, 2018; Shen et al., 2018; Wang et al., 2020). Although the number of *mcr*-positive *E. coli* genomes declined after 2016, given that some of the sequence data from 2019 to 2021 had not yet been released when we downloaded the data and conducted this study in 2021, the actual prevalence of *mcr*-positive strains worldwide should be more than the number we analyzed, so current global prevalence would likely be significantly higher. Additionally, as the poultry and swine industries accounted for 96% of total colistin sulfate livestock use (Wang et al., 2020), the presence of *mcr* in them provides evidence that colistin treatment has promoted the transmission of *mcr*, with livestock as the primary reservoir. Colistin-resistant *E. coli* can then spread by contaminating animal-derived food or contaminating crops by excrement to threaten public safety (Elbediwi et al., 2019).

The high incidence of *mcr* in our study is in accordance with a previous report (Dadashi et al., 2022), consistent with its identification as the globally predominant colistin-resistance gene. At present, *mcr*-1 could confer colistin resistance with broad minimal inhibitory concentrations (MICs) of 2–16 mg/L (Nang et al., 2019), for instance, 74% of *mcr*-1 positive *E. coli* isolates had MICs at 2 mg/L (Nguyen et al., 2022), while 52% of *mcr*-1 positive *E. coli* isolates in another study exhibited MICs of 4∼8 mg/L (Ćwiek et al., 2021). For other *mcr* genes, *mcr*-3 has been globally disseminated since it was first reported in Yin et al. (2017), and this gene inside the linker has been found to appear as a facilitator of colistin resistance (Xu et al., 2021). It also has been reported to facilitate evasion of host phagocytosis, but generally mediates low-level (≤8 mg/L) colistin resistance among *Enterobacteriaceae* (Yin et al., 2021). The *mcr-9* variant was generally susceptible to colistin with MICs of ≤1 mg/L, because it did not confer colistin resistance itself, its colistin resistance replied to gene regulation (Nakamura et al., 2021), and *mcr*-5 was demonstrated to generate colistin resistance with MICs of 4–8 mg/L in *S. enterica* (Nang et al., 2019). Notably, more than 20% *mcr*-positive strains co-carried CHßLs, mainly bla*_*NDM*_* followed by bla*_*KPC*_*, indicating that almost one-fifth of the strains in our study may belong to *CRE*. In addition, more than 50% of the strains co-carried *bla*_CTX–M_, such a high prevalence of bla*_*CTX–M*_* showed that most of these strains belonged to MDR strains. Meanwhile, the co-carriage of 16s RNA methylase, PMQRs, and fosfomycin genes suggested that the *mcr*-positive strains were also reservoirs of numerous resistance genes (Guo et al., 2022).

The VF analysis showed that almost all *mcr*-positive strains also carried *terC* and *gad* genes. To the best of our knowledge, *terC* represents one of the key proteins in tellurite and colicine resistance, implicated in phage inhibition, resistance, and pathogenicity, and is widespread among bacterial species, particularly in pathogens (Peng et al., 2021; Rodríguez-Santiago et al., 2021). In contrast, *gad* has been reported to enzymatically decrease intracellular protons linked to *E. coli* flagellar motility (Yamanaka et al., 2022) and is involved in the colonization of the gastrointestinal tract. In addition, more than half of the strains contained *iss*, *traT*, and *ompT*, which participate in resistance to serum complement, outer membrane protease, and long polar fimbriae, respectively; these affect resistance to host innate immunity and facilitate colonization and adhesion. Moreover, *iroN* was detected in approximately 25% of all isolates and has been associated with increased 30-day mortality in patients with bacteremia (Hung et al., 2019).

We identified many STs, demonstrating the diversity of *mcr*-positive strains. The high prevalence of ST10 in our study was similar to that in a previous report (Dadashi et al., 2022), however, it was different from a report that ST131 was the most common ST for extraintestinal pathogenic *E. coli* (ExPEC) strains (Manges et al., 2019). To date, *E. coli* ST10 has been shown to be a high-risk *mcr*-1-positive isolate from cattle farm environments (Ali et al., 2021); its clonal spread with ∼50% fosfomycin resistance has been found among diarrheal calves in Xinjiang province, China (He et al., 2021). It also has been isolated from poultry in Poland (Ćwiek et al., 2021). The biological costs imposed by plasmid-mediated resistance and virulence affect its survival and spread (Yang et al., 2020), thus, the relatively rare ARGs and VFs within ST10 clones may explain its rapid spread.

Curiously, STs differed from continent to continent, and as frequent carriers for *mcr*-1 and *bla*_NDM_ (Lin et al., 2020; Zhang X. et al., 2022), more ARGs and VFs were observed within the ST156 strain prevailing in Asia and Europe, indicating that it represents a major risk to public health, highlighting the need for increased surveillance. For example, *E. coli* ST156, carrying *bla*_NDM–5_, *bla*_CTX–M–65_, *bla*_OXA–10_, *bla*_TEM–1_, and *mcr*-1, has been found in clinical isolates in China (Lin et al., 2020; Zhang X. et al., 2022). Notably, ST648 has been reported as a high-risk, MDR, ESBL-producing strain in public aquatic environments (Furlan et al., 2020), but ST648 was not frequently detected in our study, with almost all of them being in North America. The differences in the distributions of ST clones may be related to antibiotic use, diet, and environmental factors. As the predominant ST among ExPEC isolates worldwide (Alangari et al., 2022), ST131 was shown to have higher virulence than other important ExPEC clones (Alqasim et al., 2020), whereas only 11 *mcr*-positive *E. coli* ST131 isolates were identified in this study, mainly from China, suggesting that ST10, rather than ST131 and ST648, is the main host for *mcr*, which may result from the fitness between the clones and *mcr* genes. Moreover, although ST216, with fewer VFs and ARGs, was dominant in Oceania, their association with IncHI2 plasmids may be of great concern (Tarabai et al., 2021).

The great diversity of plasmid replicons within *mcr*-positive strains indicates the great dissemination potential of ARGs and VFs. The high incidence of IncFIB is consistent with previous reports showing that IncFIB is common in *Enterobacteriaceae* (Khine et al., 2020). A previous study found that IncI2 and IncX conferred fitness advantages to host bacteria and outcompeted other plasmids (Bahador et al., 2018); the approximately 40% incidence of IncX and IncFII among *mcr*-positive strains suggests that most of the strains in this study have relatively high adaptability. Notably, IncI2 and IncX were common plasmids in *mcr*-1-positive strains, indicating that they may be dominant vectors driving *mcr*-1 transmission, but this finding of correlation analysis should be further confirmed. Considering the distribution deviation, we hypothesized that *mcr*-3 and *mcr*-9 were harbored by IncFII and IncHI2 plasmids, respectively, and *mcr*-5 may be borne on IncFII and IncI1-I plasmids. These findings were in accordance with previous reports that *mcr*-1 was carried by a range of plasmids such as IncHI2, IncI2, IncX4, IncFIA, and IncP (Haenni et al., 2016; Elbediwi et al., 2019; Liu et al., 2020; Sonnevend et al., 2022), and *mcr*-9 was always carried by IncHI2 (Simoni et al., 2021). Recently, *bla*_NDM_ was reported to be disseminated *via* the IncP plasmid with a broad host range (Choudhury et al., 2019); moreover, most VFs that were associated with enteroinvasive *E. coli*, such as *sit*, *iron*, *iss, iutA, ompT*, and *iroN*, were reported to be located mainly on IncFIA/FIB and Col plasmids (Touzain et al., 2018; Hayashi et al., 2019). Both *mcr*-1 and *bla*_CTX–M–1_ were colocalized on IncHI2 and IncI2 plasmids in two studies (Haenni et al., 2016; Yin et al., 2021), which support the consistent distribution of plasmids and ARGs/VFs analyzed in our study, suggesting the dissemination potential of the related ARGs and VFs.

To the best of our knowledge, ours is the first study to evaluate the prevalence of global *mcr*-harboring *E. coli* isolates using WGS data. The 778 *mcr*-positive isolates, widely distributed among the six continents during 1905–2019, could be representative of the current global *mcr*-carriers. However, this study had some limitations. First, the actual resistance phenotypes of these strains were not available for assessment of differences between genotypes and phenotypes. Second, whether the resistance and *mcr* genes were carried on a single plasmid was unclear, so we cannot clearly illustrate the specific relationships between VFs and ARGs among plasmids. Third, our study depended on the submission of genomes to the database, so it could not represent areas where no data had been uploaded.

Collectively, our data found that *mcr*-1 is the most widespread gene that confers resistance to colistin. More than 50% of the global *mcr*-positive *E. coli* isolates had numerous ARGs, and the fifth strain belonged to *CRE*. Diverse STs have been identified, further attention should be focused on the ST10 and ST156 clones, considering the rapid transmission of ST10 and the convergence of ARGs and VFs within ST156.

## Data availability statement

The original contributions presented in this study are included in this article/Supplementary material, further inquiries can be directed to the corresponding authors.

## Author contributions

XC and HS contributed to the experimental design of the study. JS, HZ, CL, HX, and CCL performed data acquisition and statistical analysis. JS, HZ, XC, and HS performed bioinformatics analysis and writing. All authors contributed to the article and approved the submitted version.

## Footnotes

1
https://www.ncbi.nlm.nih.gov/pathogens/isolates#/refgene/gene_family

2
https://cge.cbs.dtu.dk/services/PlasmidFinder/

3
https://cge.cbs.dtu.dk/services/VirulenceFinder/
